# Optimization of High-Pressure Processing for Microbial Inactivation in Pigmented Rice Grass Juice and Quality Impact Assessment during Refrigerated Storage

**DOI:** 10.3390/foods13182995

**Published:** 2024-09-21

**Authors:** Uyen Ha Dao, Jitlada Na Lamphun, Sitthidat Tongdonyod, Sirinya Taya, Suphat Phongthai, Wannaporn Klangpetch

**Affiliations:** 1 Division of Food Science and Technology, Faculty of Agro-Industry, Chiang Mai University, Chiang Mai 50100, Thailand; daohauyen@gmail.com (U.H.D.); sittidat07@gmail.com (S.T.); suphat.phongthai@cmu.ac.th (S.P.); 2 Functional Food Research Unit, Multidisciplinary Research Institute, Chiang Mai University, Chiang Mai 50200, Thailand; sirinya.t@cmu.ac.th; 3 Cluster Research of High Value Products from Thai Rice and Plants for Health, Chiang Mai University, Chiang Mai 50100, Thailand; 4 Center of Excellence in Agro Bio-Circular-Green Industry (Agro BCG), Faculty of Agro-Industry, Chiang Mai University, Chiang Mai 50100, Thailand

**Keywords:** high hydrostatic pressure, optimization, *Escherichia coli*, *Listeria innocua*, phytochemicals, cereal grass

## Abstract

Pigmented rice grass juice (RGJ) is a good source of bioactive compounds, but fresh juice has a relatively short shelf life of only 7 days at 4 °C. The objectives of this study were to determine the optimal growth stage of pigmented rice grass, investigate the optimal condition of high-pressure processing (HPP) for bacterial inactivation in inoculated RGJ using response surface methodology (RSM), and evaluate quality changes in uninoculated HPP-treated juice during storage at 4 °C compared with heat-treated (85 °C/10 min) and untreated samples. Results revealed that the optimal growth stage of rice grass was 9 days with the highest total anthocyanin content of 158.92 mg/L. The optimal condition of HPP was determined to be 612 MPa, 11 min, and 36 °C, and inactivated *Escherichia coli* K12 and *Listeria innocua* with 6.43 and 5.02 log reductions, respectively, meeting FDA regulations. The lethality of bacteria after HPP treatment can be explained by damage to the cell membrane and the leakage of intracellular constituents such as protein and nucleic acid. During 12 weeks of storage at 4 °C, total plate counts and yeast and mold counts in uninoculated HPP-treated juice were not detected. Moreover, HPP did not significantly change phytochemical properties (*p* < 0.05), caused a minor impact on physicochemical properties of RGJ, and maintained the durability of juice samples during storage. Analysis of the phytochemical profile revealed that HPP treatment could preserve most of the phenolic compounds in RGJ and especially increase the contents of protocatechuic acid, 4-hydroxybenzoic acid, syringic acid, transcinnamic acid, isorhamnetin-3-o-glucoside, quercetin, and cyanidin-3-glucoside (*p* < 0.05). Overall, HPP is a potential pasteurization technique for microbial inactivation and nutritional preservation for rice grass juice.

## 1. Introduction

Cereal grass is now considered a functional food [1]. Wheat grass juice is the most common cereal grass juice and contains considerable amounts of bioactive compounds [2]. However, due to the high price and cultivation area of wheat, products from wheat grass are not commonly marketed and consumed in Asian countries. Rice (*Oryza sativa*) belongs to the *Poaceae* family, similar to wheat, and is the most important staple food crop in Asia, which is also the leading continent in rice production, accounting for nearly 90% of the global market share based on the data of FAOSTAT (available online: https://www.fao.org/faostat/en/#data/QCL/visualize) (accessed on 9 September 2024). Kum Doi Saket (KDS) is a black-purple glutinous rice cultivar that is widely grown in the north and northeast of Thailand. Juice squeezed from rice grass harvested at the jointing stage (days 6–15 of the growth stage) exhibits the highest levels of bioactive compounds [1,3]. Therefore, the “growing stage” must be considered to maximize the phytochemical properties of cereal grass juice.

Few studies have addressed the bioactivity of rice grass juice (RGJ), with most revealing superior phytochemical characteristics in pigmented RGJ compared to wheat grass juice [4]. Khanthapoka et al. (2015) found that RGJ from a black-color cultivar exhibited the highest total phenolic content (4.3 mg GAE/g DE) and the highest radical scavenging activity (EC_50_ = 11 mg/mL) compared to other white rice and wheat grass juices [5]. Thepthanee et al. (2021) reported the cancer chemopreventive potential, strong antioxidant activity, and anti-inflammatory capacity of a black rice grass extract which was a rich source of phenolic acids, anthocyanins, and flavonoids [6]. Rice is the main staple food in Asia and black rice cultivars contain high levels of polyphenols and antioxidants. Therefore, pigmented RGJ can be developed competitively as an alternative functional beverage. RGJ is similar to wheat grass juice and is categorized as a low-acid vegetable juice with pH > 4.6 and a short shelf life [7]. Vegetable juice is defined as “the liquid unfermented but fermentable product or lactic acid fermented product intended for direct consumption obtained from the edible part of one or more sound vegetables and preserved exclusively by physical means”. The products must be free from microorganisms capable of development under normal conditions of storage [8]. Thus, finding an appropriate preservation technique is necessary to extend the shelf life of RGJ.

Today, minimally processed food products with superior quality and fresh-like characteristics are in high demand, and less harsh preservation techniques with fewer additives are preferred. Due to its advantages over thermal pasteurization, high pressure processing (HPP) has gained popularity. These benefits include (i) immediately inactivating pathogenic and spoilage-causing bacteria, (ii) delaying or deferring the commencement of chemical and enzymatic deteriorative processes, and (iii) maintaining the nutrition and organoleptic properties of food products [9,10,11,12,13].

HPP is usually applied to fruit and vegetable juices as a pasteurization method with pressures ranging from 400 to 600 MPa and treatment duration from 1 to 15 min [9]. Research has shown that HPP preserves the safety and organoleptic quality of low-acid beverages such as carrot juice [14], cucumber juice [15], wheat grass juice [16], pitaya juice [17], and coconut juice [18]. The study by Liu et al. (2016) reported that HPP (500 MPa for 5 min at 20 °C) can inactivate total aerobic bacteria and total yeast and mold counts to less than 1 log (CFU/mL) in a clear cucumber beverage, and prevent the appearance of bacterial colonies for 20 days of refrigerated storage. HPP-treated juice also retained a fresh-like color, demonstrated higher clarity, and preserved more important aroma constituents than high temperature short time (HTST)-treated juice [15].

Therefore, the objectives of this study were to determine the optimal growth stage of black rice grass, optimize the HPP conditions for inactivation of *Escherichia coli* K12 and *Listeria innocua* in Kum Doi Saket RGJ, and investigate the stability of HPP-treated RGJ during storage compared with heat-treated RGJ.

## 2. Materials and Methods

### 2.1. Study of Rice Grass Growth Stages

Kum Doi Saket black rice grass was grown by the Lanna Rice Research Center, Chiang Mai University, Chiang Mai, Thailand. The seeds were tested for >95% germination, soaked in water for 12 h and incubated for 48 h (25 °C). Then, 200 g of seeds were spread evenly on a seedling tray (30 × 60 cm) and watered regularly. Germination was conducted under controlled conditions at 25/20 °C and 60 to 70% relative humidity.

The rice grass was grown for 14 days. Harvesting began on day 6 when the grass was 12 to 15 cm high. After harvesting, the fresh grass was weighed, washed with tap water, and pretreated by soaking in sodium bicarbonate solution (0.1%, 15 min) for contaminant removal. The RGJ was then extracted by a single gear horizontal juicer (Oscar DA1200, Ulsan, Republic of Korea) and filtered through cheesecloth three times. The extracted juice was analyzed for some phytochemical properties including total phenolic content, antioxidant activity, and total anthocyanin content. Among them, total anthocyanin content was considered the most important factor. The growth stage that revealed the highest levels of bioactive contents was chosen for further experiments.

### 2.2. Optimization of Processing Conditions

#### 2.2.1. Bacterial Strain Preparation

Gram-negative bacterium *Escherichia coli* K12 (ATCC 10798) and Gram-positive bacterium *Listeria innocua* JCM 32814 were the two bacteria strains used in this study, which were provided by the American Type Culture Collection and Japan Collection of Microorganisms, respectively.

The bacterium stocks were stored at −20 °C in Tryptic Soy Broth (TSB) supplemented with 50% glycerol. Cell cultures were activated by streaking on Tryptic Soy Agar (TSA) and incubated at 37 °C for 24 h. The strains were then subcultured into 10 mL of TSB at 37 °C until they reached the early stationary phase (10^9^ CFU/mL). The cells were then washed with sterile 0.85% (*w*/*v*) NaCl solution and collected by centrifugation (6000× *g*, 10 min). The bacterial pellets were resuspended at 10^9^ CFU/mL for further study.

#### 2.2.2. Response Surface Methodology Experimental Design for Microbial Inactivation

RGJ was autoclaved at 12 °C for 15 min to inactivate all naturally occurring microorganisms in the juice. *E. coli* K12 and *L. innocua* suspensions were inoculated individually into sterile RGJ samples in 30 mL PET bottles at a concentration of 10^6^ CFU/mL. In this study, we used response surface methodology (RSM) to optimize the processing conditions for microbial inactivation. The process parameters investigated were pressure (400 to 600 MPa), time (5 to 15 min) and temperature (15 to 35 °C). The results of preliminary experiments showed that all single processing factors provided significant effects on microbial inactivation. A Box–Behnken Design (BBD) was applied with three factors including pressure, time, and temperature, coded as A, B, and C, respectively. Each factor had three levels, and the HPP condition at the center point was replicated five times. Log reduction of the two bacteria was defined as the response for the optimization process. Analysis of variance (ANOVA) was employed to evaluate the impacts of the HPP parameters on two responses with *p* < 0.05 indicative of statistically significant parameters.

After treatment, inoculated RGJ samples were investigated for the viability of bacterial cells. Appropriate decimal dilutions of cell suspensions were drop-plated onto TSA. All plates were incubated at 37 °C for 24 to 36 h. Finally, typical colonies were counted and reported as log reduction using Equation (1):

Log reduction = log(N_0_) − log (N) (1)

where N_0_ represents the count of colonies in untreated samples and N is the count of colonies in treated samples.

### 2.3. Bacterial Morphological Studies

The morphological alterations in untreated, heat-treated, and HPP-treated bacteria cells were assessed by scanning electron microscopy (SEM), based on the approach of Sha et al. (2020) with some modifications. Inoculated HPP-treated samples, heat-treated (85 °C, 10 min) samples, and untreated samples were centrifuged (6000× *g*, 10 min) to obtain the bacterial cell pellet [19]. A critical point dryer (Leica EM CPD300, Vienna, Austria) was used to dry the pellet. The dried cell was then coated with gold and subjected to SEM (JEOL, JSM-IT300, Tokyo, Japan) (15 kV) to examine the morphology [20].

### 2.4. Determination of the Intracellular Constituent Leakage

Preservative treatments like HPP or heat might cause breakage of the bacterial cell membrane resulting in the leakage of intracellular constituents. The release of nucleic acids and proteins from the two bacterial strains after treatment was determined following the method of [19]. After HPP and heat treatment, the bacterial suspension was centrifuged (6000× *g*, 10 min). The supernatant was filtered through sterile syringe filters of 0.22 µm to remove bacterial cells. Then, a UV-Vis spectrophotometer (Genesys 180, Thermo Fisher Scientific, Waltham, MA, USA) was employed to measure the absorbances of the filtered suspensions at wavelengths of 260 nm and 280 nm to examine the leakage of nucleic acid and protein, respectively [19].

### 2.5. Quality Assessment of the Treated RGJ during Storage

HPP (612 MPa, 11 min, and 36 °C)-treated samples (HPP-RGJ), heat (85 °C, 10 min)-treated samples (HT-RGJ), and control samples (C-RGJ) were evaluated for durability over 12 weeks of storage at refrigerated temperature (4 °C) based on three main attributes, including microbial, physicochemical, and phytochemical qualities.

#### 2.5.1. Microbial Quality

The microbiological quality of control, HPP-treated, and heat-treated RGJ samples during storage was evaluated by analyzing total plate count (TPCt) on plate count agar and yeast and mold count (YMC) on potato dextrose agar. Both TPCt and YMC were measured using the spread plate technique [21,22]. Finally, the samples were stored at 37 °C for 48 h. The amount of bacterial colonies found on agar was represented in log CFU/mL.

#### 2.5.2. Physicochemical Quality

The total soluble solids (TSS) was evaluated by a hand refractometer (ATAGO, Tokyo, Japan). A pH meter (FiveGo F2, Mettler Toledo, Switzerland) was employed to determine the pH value of the rice grass juice. Titratable acidity (TA) was analyzed following the methodology with some modifications [16]. Color Quest XE (Hunter Lab, Tokyo, Japan) Colorimeter was employed to measure L*, a*, and b* color parameters of RGJ.

#### 2.5.3. Phytochemical Quality

##### Total Phenolic Content (TPC)

The Folin-Ciocalteu method of Khanthapoka et al. (2015), with some modifications, was used to evaluate the TPC of rice grass juice [5]. In summary, 0.5 mL of diluted RGJ was reacted with 2.5 mL of Folin-Ciocalteu reagent 10% (*v*/*v*) (ratio 1:25) and stored in the dark for 5 min. After that, 2 mL of Na_2_CO_3_ 7.5% (*w*/*v*) was added to the reaction mixture followed by incubating in the dark for 60 min. The absorbance of the suspension was detected at 765 nm. TPC of rice grass juice was expressed as milligrams of gallic acid equivalent per liter of juice (mg GAE/L) [5].

##### Antioxidant Activity by 2,2′ Azinobis (3 Ethylbenzothiazoline 6 Sulfonic Acid) (ABTS) Assay

The ABTS value of RGJ was measured following the methodology described by Re et al. (1999) with certain changes [23]. In short, to produce the ABTS^+^ radical cation, two solutions of ABTS 7 mM and K_2_S_2_O_8_ 2.45 mM (ratio 1:1) were mixed and stored in the dark at room temperature for 16 h. Then, 3 mL of diluted ABTS^·+^ solution (absorbance of 0.7 ± 0.02 at 734 nm) was reacted with 50 µL of diluted RGJ (1:25) and incubated for 30 min in the dark at 25 °C. The absorbance was detected at 734 nm. Antioxidant activity (AOA) was defined as millimoles of Trolox equivalent per liter of juice (mmol TE/L) [23].

##### Antioxidant Activity by Ferric Reducing Antioxidant Power (FRAP) Assay

The FRAP value of RGJ was evaluated based on the study of Benzie and Strain with some changes. Briefly, FRAP reagent generated by the reaction of 300 mM acetate buffer (pH 3.6), 10 mM TPTZ, and 20 mM FeCl_3_ was reacted with 0.1 mL diluted juice (1:25). The samples were then stored in the dark for 30 min. After incubation, the absorbance of the solution was detected at 593 nm. FeSO_4_ was used to contribute the standard curve. The FRAP value of RGJ was defined in µmol Fe^2+^ per liter of juice (µmol Fe^2+^/L) [24].

##### Determination of Total Anthocyanin Content (TAC)

Anthocyanin is an important bioactive compound that contributes to the black-purple color of rice grass juice. The TAC of RGJ was investigated following the differential methodology of Lee et al. (2005) with some changes [25]. Briefly, we prepared two buffer solutions including sodium acetate 0.4 M (pH = 4.5) and potassium chloride 0.025 M (pH = 1). Then, the undiluted juice was added into each buffer solution at a ratio of 1:4 (1 part test portion, 4 parts buffer) and stored for 20 min. The absorbance of all samples was analyzed at both 520 and 700 nm. Total anthocyanin content was expressed as milligrams of cyanidin-3-glucoside equivalent per liter of juice (mg/L) and determined using Equation (2) [25]:

(2) $$\mathrm{TAC}\;\left(\mathrm{mg}/L\right)=\frac{A\times 449.2\times \mathrm{DF}\times 10^{3}}{26,900\times 1}$$

where A (absorbance) = (A_520_ − A_700_)_pH 1.0_ − (A_520_ − A_700_)_pH 4.5_; 449.2 = molecular weight of cyanidin-3-glucoside (g/mol), DF = dilution factor, 1 = pathlength (cm), 26,900 = molar extinction coefficient (L·mol^−1^·cm^−1^).

##### Determination of Total Chlorophyll Content (TCC)

Chlorophyll is also an important pigment compound found in cereal grass juice. The TCC of RGJ was investigated based on the methodology of Porra et al. (1989) with minor modifications. In short, 50 µL of RGJ was mixed with 450 µL of 80% acetone. The absorbance (A) of the sample was detected at 663 and 645 nm and TCC was determined using Equation (3) [26]:

TCC (mg/L) = 19.54A^645^ + 8.29A^663^ (3)

### 2.6. Phytochemical Profile Analysis

The phytochemical profiles (phenolic acids, flavonoids, and anthocyanin) of fresh juice (C-RGJ), HPP-treated juice (HPP-RGJ), heat-treated juice (HT-RGJ), and commercially available wheat grass juice (WGJ) and barley grass juice (BGJ) were identified and quantitated using a high-performance liquid chromatography (HPLC, Agilent 1290 Infinity II, Santa Clara, CA, USA) system with a Phenomenex C18 column (5 µm, 4.6 × 250 mm), (Torrance, CA, USA) and a diode array detector, following the methodology with some modifications [27]. Two mobile phases of acetonitrile supplemented with 0.1% (*v*/*v*) formic acid and water supplemented with 0.1% (*v*/*v*) formic acid were employed at room temperature with flow rates of 1 mL/min. The detector wavelengths were set at 280 and 320 nm [27]. There were 16 phenolic compound standards used, including gallic acid, protocatechuic acid, 4-hydroxybenzoic acid, chlorogenic acid, vanillic acid, syringic acid, p-coumaric acid, ferulic acid, transcinnamic acid, catechin, rutin, isorhamnetin-3-o-glucoside, quercetin, naringenin, cyanidin-3-glucoside, and peonidin-3-glucoside.

### 2.7. Statistical Analyses

Each experiment was conducted in triplicate. Experimental runs and data were generated and analyzed with Design-Expert version 13.0.5.0, consisting of analysis of variance (ANOVA), regression evaluation, creation of response surface plots, and prediction of the optimal conditions for HPP. Response surface methodology (RSM) was employed to optimize the process parameters (pressure, time, and temperature) of HPP. JMP version 12 software (SAS Institute, Cary, NC, USA) was used to conduct two-way ANOVA. Means were compared using Tukey’s HSD test. Significance was assigned to comparisons with a *p*-value lower than 0.05 (*p* < 0.05).

## 3. Results and Discussion

### 3.1. Effect of Rice Grass Growth Stage on Phytochemical Properties

**Figure 1:**
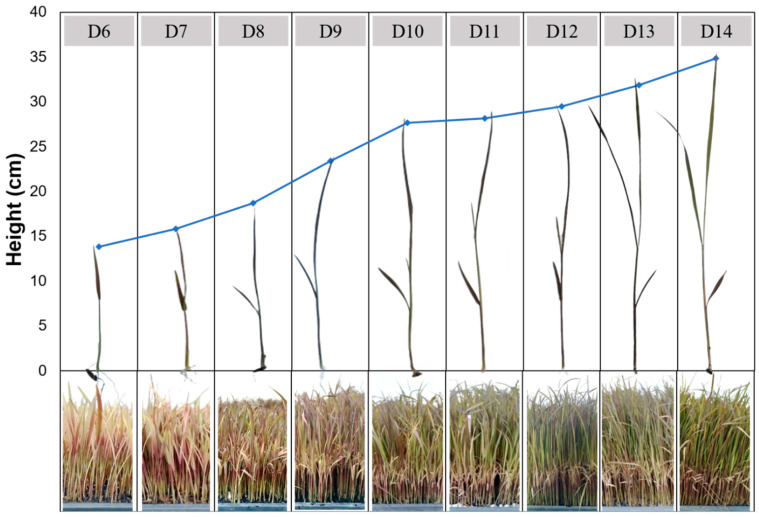
Growth stages of rice grass from day 6 to day 14.

TPC, AOA, and TAC levels in rice grass rose at 9 days after planting (*p* < 0.05) and subsequently decreased from days 11–14 (Table 1). Notably, from day 9 to day 10, TPC and ABTS values slightly increased by 7.19% and 7.57%, respectively, while FRAP value did not change significantly, but TAC decreased sharply by 22.34%. The most distinctive feature of Kum Doi Saket grass is its black-purple color, which is created by pigmented compounds such as anthocyanin. According to Khanthapoka et al. (2015), the KDS cultivar had the highest TAC compared to other cultivars of white rice grass and wheatgrass, which may result in superior antioxidant capacity and DNA protection [5]. Therefore, we considered TAC the most important parameter to determine the optimal growth stage of KDS rice grass. As a result, although the TPC and ABTS values of rice grass on day 9 were slightly lower than those on day 10, day 9 was selected as the optimal growth stage of KSD grass because TAC reached a maximum value of 158.92 mg/L. After day 9, the third leaves appeared and the color of the grass turned from dark purple to green (Figure 1), marking a gradual decrease in bioactive compounds (*p* < 0.05).

Cereal grasses after 6 to 15 days of germination are at the “jointing stage”, when they reveal the highest level of bioactive compounds [1,3]. Young plants need phytonutrients such as polyphenols or antioxidants to protect against threatening external factors [28]. After the jointing stage, bioactive compounds decline sharply as levels of cellulose increase to support the elongation of internodal tissue in the grass leaf, forming a stem [1,4]. In 2005, Randhir and Shetty discovered that polyphenols in immature maize leaves during the early germination stage were soluble. However, in the late growth stages, polyphenol concentration decreased due to lignification, which occurred when phenolic molecules were partitioned in a polymerization process to generate lignans and lignins [29]. The study of Kohler (1944) discovered that fiber content in oat leaves increased as the plant matured, but vitamins, protein, fat, and chlorophyll content declined [30].

Kapkum et al. (2011) investigated changes in TPC and AOA over 18 days of rice grass germination and found that 15 days was the optimal germination age [31]. Kulkarni et al. (2006) also found that wheat grass extract had the highest AOA after 15 days of growth. Different cereal cultivars have diverse optimal growth stages [32]. Rice grass harvested at the optimal growth stage (day 9) was used for further experiments.

### 3.2. Microbial Inactivation by HPP

#### 3.2.1. Model Fitting

Three factors of HPP including pressure, time, and temperature, along with their codes and levels, are shown in Table 2. Experimental runs generated by BBD and results of the effect of HPP parameters on bacterial inactivation are shown in Table 3. The observed responses were obtained via HPP experiments, whereas the expected responses were generated using a regression equation (Table 4).

Table 4 shows RSM plots demonstrating the effect of process factors on microbial inactivation, as well as coded values for second-order polynomial regression equations. The effects of the independent variables on the log reduction of two bacteria strains were demonstrated by the linear terms (A, B, C), quadratic terms (A^2^, B^2^, C^2^), and interaction terms (AB, AC, BC). The RSM model coefficients (R^2^) were 0.9613 and 0.9583, indicating that the generated models explained 96.13% and 95.83% of the variation for *E. coli* K12 and *L. innocua* log reduction, respectively. A model with a relatively good fit usually has a coefficient above 0.08 [33]. The findings showed that experimental and predicted values had good correlation, indicating that the quadratic models were efficacious in this research.

Analysis of variance (ANOVA) was used to examine the influence of process parameters on log reduction of the two bacteria strains, with *p*-values less than 0.05 indicating statistical significance. The data shown in Table 4 reveal that the quadratic models were significant for the optimization of HPP process parameters, with *p*-values < 0.05.

#### 3.2.2. Effect of HPP Parameters on the Inactivation of *E. coli* and *L. innocua*

**Figure 2:**
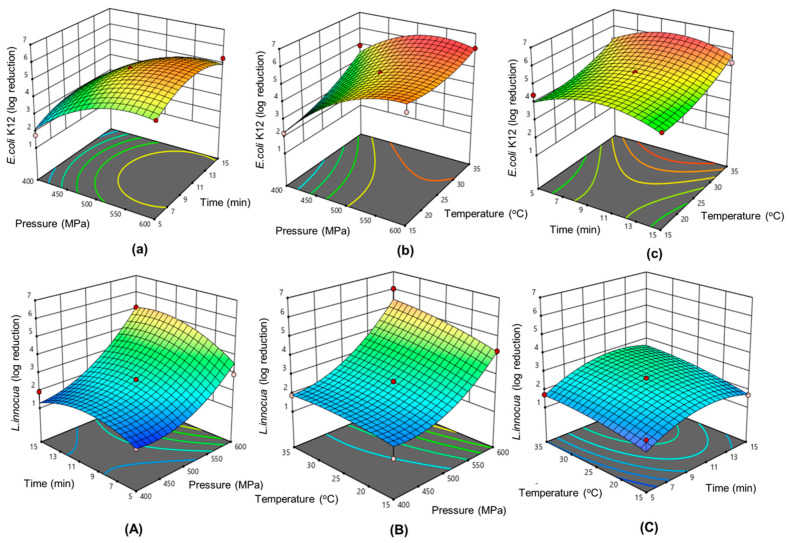
3D response surface graphs illustrating the effect of three HPP independent factors (pressure, time, temperature) on the log reduction of *E. coli* K12 (**a**–**c**) and *L. innocua* (**A**–**C**).

The three HPP processing parameters were pressure (A), processing time (B), and processing temperature (C). Figure 2 depicts response surface plots that demonstrate the correlations between the process parameters and *E. coli* K12 and *L. innocua* inactivation. Pressure caused the most significant linear effect on the log reduction of *E. coli* K12 and *L. innocua*, according to the ANOVA (Tables S3 and S4) (*p* < 0.05). At a fixed temperature of 25 °C and processing time of 15 min, an elevation of pressure from 400 to 600 MPa resulted in higher log reduction of both bacterial strains (Table 3). The ANOVA results also showed that treatment temperature was more significant than treatment time for *E. coli* K12 inactivation while the opposite results were obtained for *L. innocua*. Extension of treatment time (5 to 15 min) did not significantly affect *E. coli* K12 log reduction (*p* < 0.05).

Increased pressure (400−600 MPa) and processing temperature (15–35 °C) led to better bacterial elimination (Figure 2B,b). Moreover, the maximum pressure of 600 MPa assisted with an elevated temperature of 35 °C gave the highest inactivation of both *E. coli* K12 and *L. innocua* at 6.15 and 5.65 log reduction, respectively (Table 3). In general, HPP treatment conducted above or below room temperature (20 °C) can enhance microbe inactivation by altering bacterial strain resistance [34]. Treatment with HPP (375 MPa at 35 °C for 5 min) could increase the inactivation of *E. coli* O157:H7 by greater than 5 log reduction compared to the same HPP conditions at 25 °C [35]. Similarly, HPP-treated (400 MPa, 2 min, 20 °C) carrot juice (pH 6.4) resulted in a 3-log reduction of *L. innocua* whereas the inactivation ability was improved to more than 6 log reduction when the mild heat of 35 °C was applied [36]. These phenomena can be explained by the bacterial cells becoming more vulnerable to HPP when exposed to moderate heat due to the membrane phase transition [37].

Obviously, the resistance of the Gram-negative *E. coli* K12 and Gram-positive *L. innocua* to HPP variables differed. At all HPP conditions, the log reduction of *E. coli* K12 was higher than the log reduction of *L. innocua* (Table 3). Generally, Gram-negative bacterial cells are more vulnerable to high pressure assisted by mild heat than Gram-positive bacteria because the latter are surrounded by thicker layers of peptidoglycan [38].

Pressure was shown to be the most important parameter for the inactivation of both bacteria strains. Our experiments on sublethal injury of *E. coli* K12 and *L. innocua* after HPP treatment (Table S1) revealed that at 600 MPa and treatment time > 10 min no microbial cells were recovered on the selective medium (tryptic soy broth supplemented with 2% (*w*/*w*) sodium chloride). Increases in the injury rate of *E. coli* (89.55–100%) and *L. innocua* (78.93–100%) treated with HPP were detected when pressure increased from 400 MPa to 600 MPa. High pressure impacts microorganisms because of (i) alterations in cell morphology, including disruption of membrane permeability and loss of membrane durability; (ii) the inhibition of physiological functions and denaturation of functional proteins leading to limited proton flow, reduced intracellular pH, inactivation of enzymes responsible for synthesizing ATP, loss of functionality of membrane-bound enzymes, and disintegration of ribosomes; and (iii) the alteration of genetic systems, causing genetic materials to condense and degrade chromosomal DNA [39,40,41,42,43] The combination of high pressure and mild elevated temperature (35 °C) inactivates pathogens more effectively than pressure or temperature alone by damaging the bacterial cell membrane.

#### 3.2.3. Optimization and Verification

The FDA regulations for fruit and vegetable juice pasteurization require that preservative treatment must achieve the inactivation of pathogenic bacteria or surrogate microorganisms with more than 5 log reduction [44]. Therefore, in order to find out the predicted optimal HPP conditions, the responses for numerical optimization of HPP process parameters were set to the range of greater than 5 log reductions of both bacterial strains. Table 5 reveals the predicted optimal conditions for HPP, which was then verified by validated experiments to ensure that HPP could inactivate both bacteria to the target log reduction. As a result, the validated experimental findings varied somewhat from the predicted values. HPP treatment at 612 MPa, 11 min, and 36 °C effectively inactivated *E. coli* K12 and *L. innocua*, resulting in 6.43 and 5.02 log reduction, respectively. Similar results were also observed with pitaya juice, where the application of HPP at optimal conditions of 600 MPa for 12 min could also achieve 5 log reduction of *L. innocua* [17].

### 3.3. Mechanism of HPP on Inactivation of E. coli K12 and L. innocua

**Figure 3:**
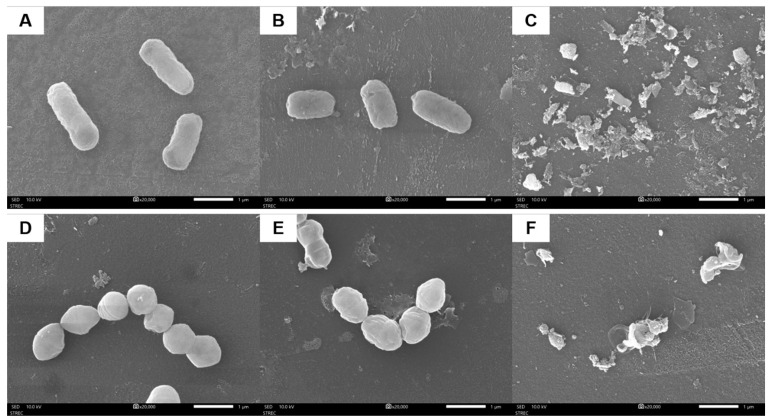
Morphology of *E. coli* K12 (**A**–**C**) and *L. innocua* (**D**–**F**) evaluated using SEM. (**A**,**D**): untreated cells; (**B**,**E**): heat-treated samples (85 °C, 10 min); (**C**,**F**): HPP-treated samples (612 MPa, 11 min and 36 °C).

The mechanism of HPP on microbial inactivation was elucidated by studying the morphology of untreated, HPP-treated, and heat-treated bacterial cells using SEM and the leakage of intracellular constituents. Figure 3 illustrates SEM micrographs of the surfaces of *E. coli* K12 and *L. innocua* cells. The cells of untreated samples (Figure 3A,D) had smooth and undamaged surfaces. By contrast, the surfaces of both strains of bacteria treated by HPP exhibited severe damage to the cell membrane (Figure 3C,F). Cells treated only with heat revealed mild defects on the surface (Figure 3B,E). Yang et al. (2012) observed cell membrane rupture in *E. coli* caused by HPP (500 MPa/30 min/25 °C) [45]. Ritz et al. (2002) reported that HPP (400 MPa, 10 °C, 20 min) induced scarring on the surface, loss of membrane integrity, and decrease in membrane functionality in *L. monocytogenes* cells [46].

**Figure 4:**
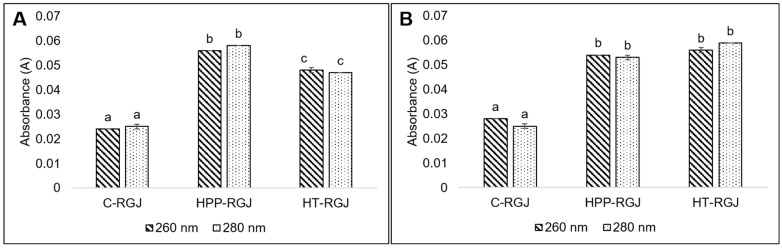
Effect of HPP (HPP-RGJ) and heat treatment (HT-RGJ) on the leakage of nucleic acid (measured at 260 nm) and protein (measured at 280 nm) of *E. coli* (**A**) and *L. innocua* (**B**) compared with the control sample (C-RGJ). Different letters indicate significantly different values among treatments (*p* < 0.05).

Cell membrane damage after heat and HPP treatment caused leakage of the intracellular constituents. UV–Vis spectrophotometry was used to evaluate the leakage of nucleic acid and protein at 260 and 280 nm, respectively (Figure 4). Differences in absorbance level between untreated and treated samples revealed the release of nucleic acid and protein through the broken cell membrane site into the supernatant. HPP caused significantly higher amounts of leaked nucleic acid compared with the heat-treated sample. These observations were consistent with the results of SEM experiments on morphology changes, which also indicated that HPP caused more severe damage to the cell membranes of both bacteria than heat treatment. Our study on sublethal injured cells of *E. coli* K12 and *L. innocua* after HPP (600 MPa, 10 min) and heat (85 °C, 10 min) treatment (Table S1) revealed that, although both these treatments were capable of achieving lethality of up to 5 log reduction, HPP treatment resulted in a higher sublethal injury ratio (%) of bacterial cells compared to heat treatment.

The results of bacterial morphology and intracellular component leakage supported the theory of HPP-mediated microbial inactivation. The structure of the bacterial cell wall is constituted of peptidoglycans, which include N-acetylglucosamine, N-acetylmuramic acid, and three amino acids (D-glutamic acid, D-alanine, and meso-diaminopimelic acid) that coat the nuclear membrane. The genetic material is vulnerable to elevated temperature and high pressure [43]. The cell membrane is the primary location of pressure injury in microorganisms [11]. Microorganism inactivation mechanisms under pressure involve modifying noncovalent electrostatic and hydrophobic interactions, resulting in the unfolding of tertiary and quaternary enzyme and protein structures that are crucial for replication, cellular integrity, and metabolism. Moreover, high pressure reduces cell volume, collapses intracellular vacuoles, and disrupts or increases membrane permeability, resulting in the loss of intracellular components like protein and nucleic acids [40].

### 3.4. Quality of RGJ during 12 Weeks of Storage

#### 3.4.1. Microbial Qualities

The mean populations of TPCt and YMC in fresh RGJ were 3.02 ± 0.01 log CFU/mL and 3.21 ± 0.04 log CFU/mL, respectively (Table 6). Immediately after HPP treatment and heat treatment, TPCt and YMC were significantly reduced below the level of detection and remained unchanged during the storage period of 12 weeks. The prolonged microbial shelf life of treated samples in this investigation can be explained by the lag phase period of bacteria increasing as well as the growth rate of injured bacterial cells decreasing [47]. Furthermore, cold storage (4 °C) postponed the commencement of the growth phase of pathogenic bacteria, induced inactivation of sub-lethally wounded cells, impeded bacterial spore germination, and prevented quality deterioration owing to residual enzymatic reactions [48,49,50,51].

Treatment with HPP (450 and 600 MPa, 5 min) resulted in the inactivation of mesophiles, yeast and molds, and lactic acid bacteria in apple juice, and the counts remained below the detection limits over 12 weeks of storage [52]. The study of Stinco et al. (2019) reported that carrot juice treated at the pressure of 450 MPa for 5 min resulted in no detection of total aerobic mesophiles and yeast and mold viable cell numbers during 14 weeks [53]. Many studies have shown that treatment by high pressure processing extended shelf life under refrigerated storage for kiwi juice (42 days) [54], grape juice (20 days) [55], cucumber juice (20 days) [15], and cloudy hawthorn berry juice (150 days) [56]. Overall, the results showed that HPP treatment improved the safety and prolonged the shelf life of RGJ at 4 °C.

#### 3.4.2. Physicochemical Qualities

**Figure 5:**
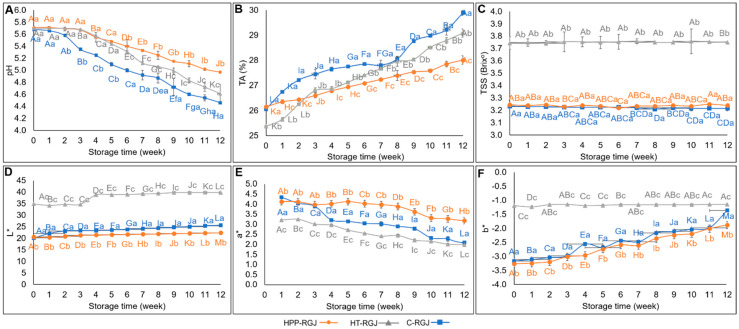
The effect of storage periods at 4 °C on pH (**A**), TA (%) (**B**), TSS (**C**), L* (**D**), a* (**E**), and b* (**F**) of HPP-treated samples (HPP-RGJ), heat-treated samples (HT-RGJ), and the control sample (C-RGJ). Different uppercase letters represent significant differences (*p* < 0.05) between storage times. Different lowercase letters represent significant differences (*p* < 0.05) between samples.

The influences of HPP and heat treatment on the stability of pH and TA values of RGJ during storage are shown in Figure 5A,B. TA (%) and pH values of the control RGJ samples were 26.05 ± 0.07 and 5.68 ± 0.01, respectively. These values did not significantly change after HPP treatment while thermal treatment increased TA and decreased pH values. The research on wheat grass juice and on cucumber juice also reported that pH and TA remained unchanged after HPP treatment [15,57]. HPP maintained the pH of juice samples for 3 weeks of storage. The overall trend of sample pH values was downward, whereas TA (%) increased gradually during the storage period. At week 12, the acidity (%) of untreated, HPP-treated, and heat-treated samples had increased by 12.84%, 6.64%, and 12.78%, respectively. Fresh cucumber juice had a similar pH to RGJ at 5.7. The study of Liu et al. (2016) also reported that HPP (500 MPa, 5 min) and high temperature short time (110 °C, 8.6 s) decreased pH and increased TA in cucumber juice after 20 days of refrigeration [15]. Similar findings on cucumber juice also revealed that the pH values of HPP-treated (400 MPa, 4 min) and heat-treated (85 °C, 15 s) juice samples decreased with refrigeration [58]. Depending on the nature of the juice matrix and pressure levels applied, HPP caused the ionization of small molecules like H_2_O and weak acids, resulting in the release of hydrogenic ions and increased juice acidity. Thus, compression of the foodstuff influenced the pH and acidity levels [12,59].

TSS did not vary significantly across samples throughout the 12 weeks of storage (*p* > 0.05) (Figure 5C). Liu et al. (2016) found that HPP and HTST treatment resulted in no significant influence on the TSS value of clear cucumber juice and TSS of all samples remained unchanged during storage (4 °C) [15]. The result is also consistent with research on cloudy cucumber juice [58] and clear apple juice [60].

**Figure 6:**
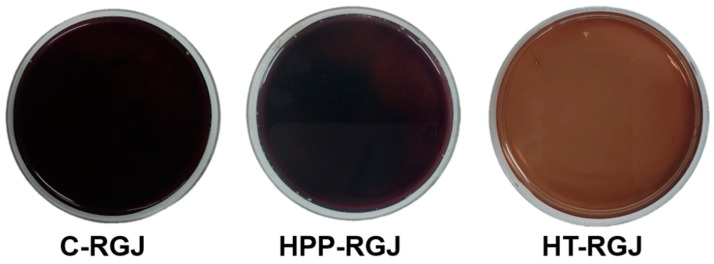
Color of HPP-treated sample (HPP-RGJ), heat-treated sample (HT-RGJ), and control sample (C-RGJ).

The color of fruit juices is a key factor in determining consumer sensory acceptability and marketability. The alteration in color parameters (L*, a*, and b*) of HPP and heat-treated RGJ during 12 weeks of storage were evaluated (Figure 5D–F). L*, a*, and b* values of the control RGJ samples were 20.14 ± 0.01, 4.34 ± 0.02, and –3.16 ± 0.02, respectively. After treatment, thermal pasteurization caused changes in RGJ color by elevating the L* and b* values and decreasing the a* value, turning the juice color from black-purple to brown (Figure 6). By contrast, HPP treatment decreased L*, a*, and b* values. Throughout the storage period, the L* and b* values of all samples gradually increased while the a* value decreased. Similar L* value increases were observed in carrot juice during storage (4 °C) [61] and in mulberry juices after thermal and HPP treatment [62]. The increase in L* values was caused by the expulsion of air from the pulp tissue due to cell disruption by pressurization or thermal action, thus making the juice lighter and more opaque [63,64]. All treatments resulted in an increase in the b* value (increase in yellowness) and decrease in a* value (decrease in redness). These results concurred with studies on jabuticaba juice [65] and cucumber juice [15]. Color changes in HPP-treated samples during storage might be induced by the activities of PPO and POD enzymes, which cause discoloration in polyphenols [63]. Overall, HPP preserved the natural color of RGJ after treatment and minimized color changes during storage. A study on pineapple juice reported that HHP had a minor influence on the color of fruit products [66].

#### 3.4.3. Phytochemical Qualities

**Figure 7:**
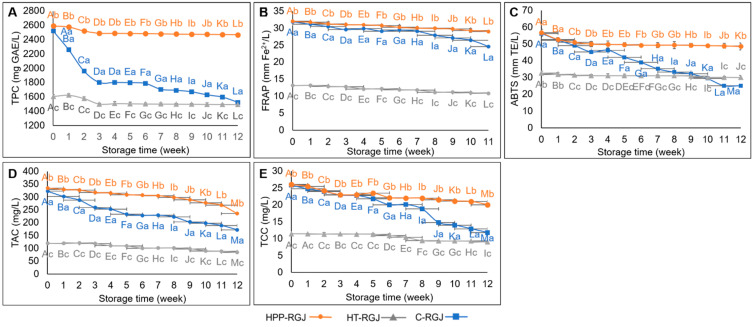
The effect of storage period at 4 °C on TPC (**A**), FRAP (**B**), ABTS (**C**), TAC (**D**), and TCC (**E**) of the HPP-treated sample (HPP-RGJ), the heat-treated sample (HT-RGJ), and the control sample (C-RGJ). Different uppercase letters represent significant differences (*p* < 0.05) between storage times. Different lowercase letters represent significant differences (*p* < 0.05) between samples.

Rice grass juice (RGJ) is a good source of bioactive components. The TPC, FRAP, and ABTS values of initial RGJ samples were 2520.24 ± 67.44 (mgGAE/L), 31.87 ± 0.35 (mmFe^2+^/L), and 56.28 ± 0.17 (mmTE/L), respectively. As shown in Figure 7, thermal treatment had an intense impact on phenolic compounds and antioxidants in RGJ by decreasing TPC, FRAP, and ABTS values by 36.23%, 41.20%, and 41.93%, respectively. By contrast, treatment with high pressure from 400 to 600 MPa did not significantly affect the ABTS value and slightly increased TPC in RGJ.

Black rice grass cultivars have high contents of pigmented compounds. The TAC and TCC of fresh RGJ were 322.47 ± 2.76 mg/L and 25.82 ± 0.16 mg/L, respectively (Figure 7D,E). Concurring with color changes in heat-treated RGJ described previously, thermal treatment significantly decreased both TAC and TCC by 62.89% and 55.77%, respectively. The HPP-treated samples recorded an increase in TAC and TCC compared with the control sample, consistent with the increase in TPC after HPP treatment. No significant effects of HPP treatments on cyanidin-3-glucoside content were reported compared with the control, with over 98% of cyanidin-3-glucoside content retained [67].

The increase in TPC, TAC, and TCC was related to the improved extractability of bioactive components. Based on Le Chatelier’s theory, a system’s volume decreases throughout the pressure-promoting period. HPP promoted solvent penetration that integrated with bioactive components by breaking the cell membranes and accelerating mass transfer by increasing membrane permeability [68,69]. Based on the study of Lou et al. (2022), HPP treatment made the microstructure of the cloudy hawthorn berry juice matrix looser, wrinkled, porous, and fractured, boosting the rate of phenolic compound extraction. They also explained that the phenolic increase following HPP treatment was mostly attributable to an increase in procyanidins and flavonoids [70].

The preservation effect of HPP on bioactive compounds can be explained by two causes. High pressure from 400 to 600 MPa only impacts non-covalent bonds, with little effect on phytochemical constituents that impart flavor, color, and nutritional compounds, while uniform pressure is instantaneously transmitted across all dimensions of the food products, regardless of their size and geometry [9,71].

During storage at 4 °C, the bioactive compound levels of fresh juice samples gradually degraded, while HPP and heat treatment slowed down the degradation of phytochemical constituents and maintained TPC, AOA, TAC, and TCC values due to effective enzyme inactivation. Results of the enzyme inactivation experiments (Table S2) indicated that HPP inactivated peroxidase (POD) and polyphenol oxidase (PPO) in RGJ with decreases of 38.96% and 23.08%, respectively, while heat treatment completely inactivated enzyme activities. A study on cloudy apple juice indicated that the optimal pressure was 600 MPa to retain the content of flavonoids and other polyphenols while reducing the activity of polyphenoloxidase and peroxidase [72].

### 3.5. Phytochemical Profiles

The phytochemical profiles of the control sample (C-RGJ), HPP-treated sample (HPP-RGJ), and heat-treated sample (HT-RGJ) were compared with commercially available wheat grass juice (WGJ) and barley grass juice (BGJ) samples (Table 7). Results showed that RGJ was a good source of phytochemical compounds. Compared to fresh RGJ, heat treatment caused significant degradation of the 11 phenolic compounds. HPP application preserved most of the contained compounds and also increased the contents of some polyphenols including protocatechuic acid, 4-hydroxybenzoic acid, syringic acid, transcinnamic acid, isorhamnetin-3-o-glucoside, quercetin, and cyanidin-3-glucoside compared with the control samples, consistent with the increases in TPC and TAC in the HPP-treated samples mentioned previously. HPP promoted increased concentrations of some polyphenols in cloudy apple juice such as p-coumaroylquinic acid, caffeoylquinic acid isomer, chlorogenic acid, (-)-epicatechin, phloridzin, phloretin 2-o-xylosylglucoside, procyanidin B2, and procyanidin C1. Improved extraction and determination of bioactive compounds after HPP treatment were achieved because the high pressure broke the cell membrane, leading to better solvent penetration and membrane permeability [56,73].

The commercially available wheat grass juice and barley grass juice samples did not contain catechin, isorhamnetin-3-o-glucoside, naringenin, or two anthocyanin compounds including cyanidin-3-glucoside and peonidin-3-glucoside. Black RGJ revealed a 2 to 10-fold higher content of most phytochemicals determined in this study compared to the two commercial products. This result concurred with Khanthapoka et al. (2015) who reported that RGJ from a black-color rice cultivar exhibited higher TPC and AOA compared to a wheat grass juice sample [5].

## 4. Conclusions

High pressure processing is an innovative non-thermal processing technique which is useful for industrial processing of fruit and vegetable juices. This study reports that HPP at 612 MPa, 11 min and 36 °C was determined to be the optimal condition to inactivate *E. coli* and *L. innocua* for more than 5 log reduction in RGJ, meeting the regulations of the FDA. HPP treatment at the optimal condition could prolong the microbial shelf life of RGJ by at least 12 weeks. This technique also preserved both the physicochemical and phytochemical properties during storage at 4 °C, unlike heat treatment (85 °C/10 min). Moreover, HPP-treated RGJ contained significantly higher phytochemical amounts compared with commercially available wheat grass juice and barley grass juice. The results reveal that high pressure processing is a potential alternative to thermal pasteurization in improving the shelf life and preserving the nutritional quality of low-acidity juice. Therefore, this finding suggests the possibility of developing a new functional cereal beverage preserved by HPP from pigmented rice grass. However, incomplete enzyme inactivation was the limitation of HPP, which caused the deterioration of bioactive compounds during long-term storage. Thus, more in-depth study on this issue is necessary in the future.

## Figures and Tables

**Table 1:** Phytochemical properties of RGJ at 6 to 14 days of growth.

Attributes	Growth Stage (Days)								
	6	7	8	9	10	11	12	13	14
TPC (mg GAE/L)	3092.99 ± 156.28 ^de^	3349.02 ± 81.71 ^c^	3568.65 ± 18.57 ^b^	3433.11 ± 161.34 ^bc^	3680.70 ± 49.14 ^a^	3390.44 ± 179.1 ^bc^	3263.68 ± 30.43 ^cd^	3010.16 ± 104 ^e^	3148.22 ± 32.46 ^de^
FRAP (mm Fe^2+^/L)	19.79 ± 0.57 ^e^	22.99 ± 0.52 ^d^	29.76 ± 1.09 ^b^	31.76 ± 1.61 ^a^	32.58 ± 0.77 ^a^	25.98 ± 1.78 ^c^	25.45 ± 0.43 ^c^	25.54 ± 1.11 ^c^	27.35 ± 1.55 ^c^
ABTS (mm TE/L)	26.28 ± 1.87 ^de^	27.38 ± 2.5 ^de^	32.47 ± 3.17 ^b^	31.71 ± 1.86 ^bc^	34.11 ± 2.14 ^a^	28.21 ± 1.09 ^cd^	25.73 ± 2.47 ^de^	24.36 ± 1.95 ^ef^	20.92 ± 1.56 ^f^
TAC (mg/L)	68.97 ± 3.28 ^d^	77.65 ± 1.76 ^d^	87.11 ± 9.16 ^c^	158.92 ± 3.2 ^a^	123.41 ± 3.17 ^b^	91.4 ± 7.52 ^c^	95.13 ± 4.83 ^c^	87.84 ± 3.78 ^c^	95.46 ± 5.21 ^c^

Different superscripts represent significant differences (*p* < 0.05).

**Table 2:** Three independent variables (pressure, time, temperature) used for optimization of HPP via Box–Behnken Design (BBD).

Independent Variable (Factor)	Label	Level		
		−1	0	−1
Pressure (MPa)	A	400	500	600
Time (h)	B	5	10	15
Temperature (°C)	C	15	25	3

**Table 3:** The impact of independent HPP factors (pressure, time, temperature) on the inactivation of *E. coli* K12 and *L. innocua*.

Run	Factors			Response (*E. coli* K12)		Response (*L. innocua*)	
	A: Pressure (MPa)	B: Time (min)	C: Temperature (°C)	Actual	Predicted	Actual	Predicted
1	600	10	35	6.15	6.25	5.65	5.12
2	600	15	25	5.30	5.05	5.09	4.96
3	600	10	15	5.24	5.77	4.25	3.90
4	500	15	35	5.24	5.75	2.99	3.18
5	500	10	25	5.18	5.27	2.64	2.52
6	500	10	25	5.18	5.27	2.64	2.52
7	500	10	25	5.18	5.27	2.64	2.52
8	500	10	25	5.18	5.27	2.64	2.52
9	500	5	35	5.18	5.30	1.97	1.70
10	500	10	25	5.18	5.20	2.64	2.52
11	400	10	35	4.93	4.57	1.88	1.98
12	600	5	25	4.56	4.64	2.92	3.25
13	500	5	15	4.43	4.10	1.71	1.28
14	500	15	15	4.26	4.26	1.70	1.73
15	400	15	25	2.24	2.33	1.94	1.36
16	400	10	15	2.21	2.29	1.05	1.33
17	400	5	25	1.76	2.19	1.26	1.14

**Table 4:** Second-order polynomial regression equations for microbial inactivation by HPP.

	*E. coli* K12	*L. innocua*
R^2^	0.9613	0.9583
*p*-value	0.0004 (significant)	0.0005 (significant)
Equation	Y_1_ = 5.18 + 1.27A + 0.1388B + 0.6701C − 0.0680AB − 0.4538AC + 0.0585BC − 0.9316A^2^ − 0.7849B^2^ + 0.3824C^2^	Y_2_ = 2.64 + 1.47A + 0.4820B + 0.4701C + 0.3727AB + 0.1440AC +0.2582BC + 0.6345A^2^ − 0.4788B^2^ − 0.0715C^2^

**Table 5:** Predicted and validated values for bacterial inactivation.

Value	Optimized Process Parameters			Responses	
	A: Pressure (P)	B: Time (min)	C: Temperature (°C)	*E. coli* K12 log Reduction	*L. innocua* log Reduction
Predicted	612.00	11.2	36.25	6.25	5.12
Validated	612.00	11.00	36.00	6.43	5.02
% error	0.00	1.78	0.69	1.42	1.95

**Table 6:** Changes in the microbial population in untreated, HPP-treated, and heat-treated RGJ during storage at 4 °C.

Week	TPCt (log CFU/mL)			YMC (log CFU/mL)		
	C-RGJ	HPP-RGJ	HT-RGJ	C-RGJ	HPP-RGJ	HT-RGJ
0	3.02 ± 0.01 ^j^	n.d	n.d	3.21 ± 0.04 ^k^	n.d	n.d
1	3.77 ± 0.02 ^h^	n.d	n.d	3.28 ± 0.01 _j_	n.d	n.d
2	4.91 ± 0.01 ^d^	n.d	n.d	5.50 ± 0.01 ^e^	n.d	n.d
3	5.67 ± 0.04 ^a^	n.d	n.d	6.50 ± 0.02 ^c^	n.d	n.d
4	5.66 ± 0.02 ^a^	n.d	n.d	6.78 ± 0.03 ^a^	n.d	n.d
5	5.50 ± 0.03 ^c^	n.d	n.d	6.77 ± 0.04 ^a^	n.d	n.d
6	5.51 ± 0.01 ^c^	n.d	n.d	6.70 ± 0.05 ^b^	n.d	n.d
7	5.55 ± 0.05 ^b^	n.d	n.d	6.71 ± 0.01 ^b^	n.d	n.d
8	4.89 ± 0.01 ^e^	n.d	n.d	6.02 ± 0.07 ^d^	n.d	n.d
9	4.88 ± 0.01 ^f^	n.d	n.d	5.22 ± 0.01 ^f^	n.d	n.d
10	4.55 ± 0.01 ^g^	n.d	n.d	5.03 ± 0.01 ^g^	n.d	n.d
11	3.28 ± 0.09 ^i^	n.d	n.d	4.32 ± 0.01 ^h^	n.d	n.d
12	3.01 ± 0.02 ^j^	n.d	n.d	4.21 ± 0.11 ^i^	n.d	n.d

C-RGJ: control sample; HPP-RGJ: HPP-treated sample; HT-RGJ: heat-treated sample. n.d.: not detected. Different superscripts represent significant differences (*p* < 0.05).

**Table 7:** Phytochemical profiles of the control sample (C-RGJ), HPP-treated sample (HPP-RGJ), and heat-treated sample (HT-RGJ) compared with commercially available wheat grass juice (WGJ) and barley grass juice (BGJ).

Phenolic Compound	C-RGJ	HPP-RGJ	HT-RGJ	WGJ	BGJ
Gallic acid	22.07 ± 0.02 ^a^	18.94 ± 0.01 ^b^	2.97 ± 0.03 ^c^	2.13 ± 0.01 ^d^	1.84 ± 0.02 ^e^
Protocatechuic acid	6.69 ± 0.21 ^a^	9.26 ± 0.01 ^b^	6.76 ± 0.01 ^c^	0.65 ± 0.03 ^d^	0.55 ± 0.02 ^e^
4-Hydroxybenzoic acid	12.36 ± 0.02 ^a^	10.91 ± 0.01 ^b^	1.83 ± 0.01 ^c^	0.64 ± 0.22 ^d^	0.96 ± 0.01 ^e^
Chlorogenic acid	26.74 ± 0.03 ^a^	25.74 ± 0.03 ^b^	14.33 ± 0.04 ^c^	13.25 ± 0.01 ^d^	12.87 ± 0.07 ^e^
Vanillic acid	13.1 ± 0.11 ^a^	14.52 ± 0.02 ^b^	1.00 ± 0.01 ^c^	0.39 ± 0.02 ^d^	0.22 ± 0.01 ^e^
Syringic acid	10.14 ± 0.02 ^a^	146.57 ± 0.01 ^b^	146.99 ± 0.07 ^c^	68.49 ± 0.06 ^d^	39.05 ± 0.01 ^e^
p-coumaric acid	686.57 ± 0.01 ^a^	673.32 ± 0.01 ^b^	12.67 ± 0.01 ^c^	n.d	9.61 ± 0.01 ^e^
Ferulic acid	254.59 ± 0.02 ^a^	254.64 ± 0.01 ^a^	35.97 ± 0.02 ^b^	4.53 ± 0.03 ^c^	6.5 ± 0.01 ^d^
Transcinnamic acid	1.3 ± 0.02 ^bc^	3.43 ± 0.02 ^a^	1.25 ± 0.01 ^c^	2.52 ± 0.22 ^ab^	2.07 ± 0.04 ^ab^
Catechin	7.33 ± 0.02 ^a^	12.22 ± 0.01 ^b^	51.38 ± 0.02 ^c^	n.d	n.d
Rutin	10.12 ± 0.02 ^a^	11.22 ± 0.03 ^b^	137.31 ± 0.02 ^c^	2.93 ± 0.02 ^d^	3.64 ± 0.01 ^e^
Isorhamnetin-3-o-glucoside	27.95 ± 0.01 ^a^	102.02 ± 0.07 ^b^	100.14 ± 0.02 ^c^	n.d	n.d
Quercetin	9.85 ± 0.01 ^a^	11.43 ± 0.01 ^b^	2.03 ± 0.05 ^c^	4.12 ± 0.01 ^d^	n.d
Naringenin	115.81 ± 0.07 ^a^	88.79 ± 0.02 ^b^	39.04 ± 0.13 ^c^	137.03 ± 0.12 ^d^	0.59 ± 0.01 ^e^
Cyanidin-3-glucoside	2.26 ± 0.05 ^a^	7.01 ± 0.04 ^b^	0.73 ± 0.01 ^b^	n.d	n.d
Peonidin-3-glucoside	n.d	n.d	n.d	n.d	n.d

Different superscripts represent significant differences (*p* < 0.05). n.d: not detected.

## Data Availability

The original contributions presented in the study are included in the article; further enquiries can be directed to the corresponding authors.

## Supplementary Materials

The following supporting information can be downloaded at: https://www.mdpi.com/article/10.3390/foods13182995/s1, Table S1: Sublethal injury ratio (%) of *E. coli* and *L. innocua* in HT-treated and HPP-treated RGJ; Table S2: Changes in enzyme activity in untreated, HPP-treated, and heat-treated RGJ during storage at 4 °C; Table S3: ANOVA of the effect of HPP variables on *E. coli* K12 log reduction; Table S4: ANOVA of the effect of HPP variables on *L. innocua* log reduction.
