# Supplementary material for: Optimization of High-Pressure Processing for Microbial Inactivation in Pigmented Rice Grass Juice and Quality Impact Assessment during Refrigerated Storage
Source: Foods. 2024 Sep 21;13(18):2995. doi: 10.3390/foods13182995 (PMC11431240; doi:10.3390/foods13182995)
Supplement: Supplementary file 1 [file foods-13-02995-s001.zip › foods-3198820-supplementary.pdf]

SUPPLEMENTARY DATA

S1. Determination of injured cells after treated by heat treatment (HT) and high pressure processing (HPP)

The effects of HT and HPP on *E. coli and L. innocua* cells were determined by comparing the survival levels of the samples plated onto nonselective (Tryptic Soy Agar) and selective media (Tryptic Soy Agar supplementing with 2% NaCl) after exposure to high temperature (80ºC/2-8 min) or high pressure (400– 600 MPa/10 min) (**Table S1**). On the other word, the sublethal ratio (%) expressed the percentage of injured cells which can grow on non-selective medium (TSA) but cannot recovered on selective one.

Table S1. Sublethal ratio (%) of *E. coli* and *L. innocua* in HT-treated and HPP-treated RGJ.

	Treatment condition	Sublethal ratio (%) of *E. coli*	Sublethal ratio (%) of *L. innocua*
HT	80ºC/2min	32.89 ± 0.77^{a}	35.13 ± 5.22^{a}
	80ºC/4min	33.23 ± 3.18^{a}	54.47 ± 2.32^{b}
	80ºC/6min	67.22 ± 2.45^{b}	61.62 ± 0.94^{c}
	80ºC/8min	72.22 ± 4.81^{b}	76.11 ± 3.47^{d}
HPP	400 MPa/10min	89.55 ± 0.096^{A}	78.93 ± 0.07^{A}
	500 MPa/10min	100.00 ± 0.00^{B}	91.28 ± 2.22^{B}
	600 MPa/10min	100.00 ± 0.00^{B}	100.00 ± 0.00^{C}

Different letters represented a significant difference ($p < 0.05$)

S2. Effect of heat treatment (HT) and high pressure processing (HPP) on enzyme inactivation in rice grass juice

HPP-treated sample (612MPa, 11 min, and 36 °C) and heat-treated sample (85 °C, 10min) were evaluated for the change in activity of peroxidase (POD) and polyphenol oxidase (PPO) enzyme during 12 weeks of storage at 4 °C. The results were shown in **Table S2**.

Table S2. Changes in the enzyme activity in untreated, HPP-treated, and heat-treated RGJ during storage at 4 °C.

Storage time (week)	POD (Abs/min)			PPO (Abs/min)		
	C-RGJ	HPP-RGJ	HT-RGJ	C-RGJ	HPP-RGJ	HT-RGJ
0	0.77 ± 0.04^{Aa}	0.47 ± 0.02^{Ab}	0.02 ± 0.01^{Ac}	0.13 ± 0.02Aa	0.10 ± 0.02Ab	0.01 ± 0.02Ac
1	0.89 ± 0.05^{Ba}	0.52 ± 0.01^{Bb}	0.03 ± 0.00^{Bc}	0.15 ± 0.00Ba	0.11 ± 0.01Bb	0.00 ± 0.00Bc
2	1.02 ± 0.01^{Ca}	0.55 ± 0.01^{Cb}	0.00 ± 0.00^{Cc}	0.18 ± 0.04Ca	0.11 ± 0.00Bb	0.00 ± 0.00Bc
3	1.19 ± 0.02^{Da}	0.57 ± 0.05^{Cb}	0.00 ± 0.00^{Cc}	0.22 ± 0.01Da	0.12 ± 0.02Cb	0.00 ± 0.00Bc
4	0.77 ± 0.05^{Ea}	0.47 ± 0.03^{Db}	0.00 ± 0.00^{Cc}	0.13 ± 0.0Ea	0.10 ± 0.03Db	0.00 ± 0.00Bc
5	0.59 ± 0.06^{Fa}	0.32 ± 0.02^{Eb}	0.00 ± 0.00^{Cc}	0.03 ± 0.01Fa	0.01 ± 0.01Eb	0.00 ± 0.00Bc
6	0.41 ± 0.02^{Ga}	0.25 ± 0.02^{Fb}	0.00 ± 0.00^{Cc}	0.00 ± 0.00Ga	0.00 ± 0.00Fa	0.00 ± 0.00Ba
7	0.33 ± 0.03^{Ha}	0.17 ± 0.06^{Gb}	0.00 ± 0.00^{Cc}	0.00 ± 0.00Ga	0.00 ± 0.00Fa	0.00 ± 0.00Ba
8	0.37 ± 0.06^{Ia}	0.02 ± 0.04^{Hb}	0.00 ± 0.00^{Cc}	0.00 ± 0.00Ga	0.00 ± 0.00Fa	0.00 ± 0.00Ba
9	0.27 ± 0.07^{Ja}	0.00 ± 0.00^{Hb}	0.00 ± 0.00^{Cc}	0.00 ± 0.00Ga	0.00 ± 0.00Fa	0.00 ± 0.00Ba
10	0.12 ± 0.03^{Ka}	0.00 ± 0.00^{Hb}	0.00 ± 0.00^{Cc}	0.00 ± 0.00Ga	0.00 ± 0.00Fa	0.00 ± 0.00Ba
11	0.02 ± 0.04^{La}	0.00 ± 0.00^{Hb}	0.00 ± 0.00^{Cc}	0.00 ± 0.00Ga	0.00 ± 0.00Fa	0.00 ± 0.00Ba
12	0.02 ± 0.07^{La}	0.00 ± 0.00^{Hb}	0.00 ± 0.00^{Cc}	0.00 ± 0.00Ga	0.00 ± 0.00Fa	0.00 ± 0.00Ba

C-RGJ: control sample; HPP-RGJ: HPP-treated sample; HT-RGJ: heat-treated sample
Different uppercase letters represent significant differences ($p < 0.05$) between storage times. Different lowercase letters represent significant differences ($p < 0.05$) between samples.

S3. ANOVA analysis of the effect of HPP variables on microbial inactivation

Analysis of variance (ANOVA) was performed to determine the effect of the process parameters (pressure, time, temperature) on the log reduction of *E.coli* K12 and *L.innocua*, with p-values below 0.05 indicative of statistical significance. The results were shown in **Table S3** and **Table S4**

Table S3. ANOVA analysis of the effect of HPP variables on *E. coli* K12 log reduction

Source	Sum of Squares	df	Mean Square	F-value	p-value	
**Model**	24.37	9	2.71	19.40	0.0004	significant
A-pressure	12.83	1	12.83	91.88	< 0.0001	
B-time	0.1540	1	0.1540	1.10	0.3285	
C-temperature	3.59	1	3.59	25.71	0.0014	
AB	0.0185	1	0.0185	0.1325	0.7266	
AC	0.8263	1	0.8263	5.92	0.0452	
BC	0.0137	1	0.0137	0.0981	0.7633	
A^2	3.65	1	3.65	26.16	0.0014	
B^2	2.60	1	2.60	18.60	0.0035	
C^2	0.6168	1	0.6168	4.42	0.0736	

Table S4. ANOVA analysis of the effect of HPP variables on *L. innocua* log reduction

Source	Sum of Squares	df	Mean Square	F-value	p-value	
**Model**	26.38	9	2.93	8.85	0.0044	significant
A-pressure	18.50	1	18.50	55.88	0.0001	
B-time	1.21	1	1.21	3.64	0.0980	
C-temperature	1.05	1	1.05	3.16	0.1185	
AB	0.5558	1	0.5558	1.68	0.2362	
AC	0.2304	1	0.2304	0.6958	0.4317	
BC	0.0206	1	0.0206	0.0622	0.8102	
A^2	2.97	1	2.97	8.96	0.0201	
B^2	1.97	1	1.97	5.94	0.0449	
C^2	0.1364	1	0.1364	0.4120	0.5414	
